# The Amino-Proximal Region of the Coat Protein of Cucumber Vein Yellowing Virus (Family *Potyviridae*) Affects the Infection Process and Whitefly Transmission

**DOI:** 10.3390/plants10122771

**Published:** 2021-12-15

**Authors:** Svenja Lindenau, Stephan Winter, Paolo Margaria

**Affiliations:** Plant Virus Department, Leibniz Institute DSMZ-German Collection of Microorganisms and Cell Cultures, 38124 Braunschweig, Germany; svenja.lindenau@hotmail.de (S.L.); stephan.winter@dsmz.de (S.W.)

**Keywords:** *Ipomovirus*, capsid protein, *Bemisia tabaci*, plant-virus-vector interactions

## Abstract

Most plant viruses rely on vector transmission for their spread and specific interactions between vector and virus have evolved to regulate this relationship. The whitefly *Bemisia tabaci*- transmitted cucumber vein yellowing virus (CVYV; genus *Ipomovirus*, family *Potyviridae*) is endemic in the Mediterranean Basin, where it causes significant losses in cucurbit crops. In this study, the role of the coat protein (CP) of CVYV for *B. tabaci* transmission and plant infection was investigated using a cloned and infectious CVYV cDNA and a collection of point and deletion mutants derived from this clone. Whitefly transmission of CVYV was abolished in a deletion mutant lacking amino acids in position 93–105 of the CP. This deletion mutant caused more severe disease symptoms compared to the cDNA clone representing the wild-type (wt) virus and movement efficiency was likewise affected. Two virus mutants carrying a partially restored CP were transmissible and showed symptoms comparable to the wt virus. Collectively, our data demonstrate that the N-terminus of the CVYV CP is a determinant for transmission by the whitefly vector and is involved in plant infection and symptom expression.

## 1. Introduction

Cucumber vein yellowing virus (CVYV) causes significant damage to cucumber and other cucurbit crops, such as zucchini and melon. The virus was first described in 1960 in Israel [1], and after its first finding it has now been reported in the Middle-East, the Mediterranean basin and Sudan [2,3,4,5,6,7,8,9,10]. Infected cucumber plants show netting and vein yellowing on young leaves, while older leaves show a general chlorosis [11]. Plant growth is compromised, flowers are aborted, and less fruits develop. The fruits can show a yellow/green mosaic pattern and taste dull, thus making them unmarketable.

**Figure 1:**
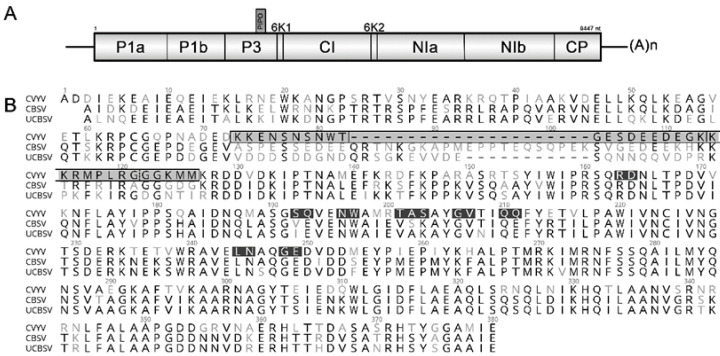
Schematic representation of the genome of cucumber vein yellowing virus (CVYV) and coat protein (CP) amino acid residues targeted for mutagenesis. (**A**) Genomic organization of CVYV isolate DSMZ PV-0776. (**B**) Alignment of CP amino acid sequences: CVYV (isolate DSMZ PV-0776), CBSV (GenBank acc. NC_012698) and UCBSV (acc. NC_014791). A total of 13 mutants were generated, including 8 alanine-substitution mutants (black), 3 deletion mutants (gray region marked by a black box; vertical lines separate the three deleted regions) and 2 partially reverted mutants in the Del3 region (the reverted regions, 8 and 5 amino acids in length, are separated by a dotted line).

CVYV belongs to the genus *Ipomovirus* (family *Potyviridae*) [12]. The filamentous particles contain a single-stranded positive-sense RNA genome, ~9.4 kb in length, with a single large open reading frame (ORF) encoding a polyprotein of ~3100 amino acids, which is proteolytically processed by virus-encoded proteinases to yield the mature viral proteins [13,14,15] (Figure 1A). The genomes of *Ipomoviruses* are distinct from other viruses in the family *Potyviridae* and virus species in this genus have unique genome arrangements [16]. The CVYV genome lacks a sequence coding for a putative HC-Pro. Instead, the genome contains two copies of P1 (P1a/P1b) and P1b carries the silencing suppression function [17].

CVYV is transmitted in a semi-persistent manner by *Bemisia tabaci* whiteflies [11,18]. For this important biological function, coat proteins (CP), besides being structural components of the viral capsid and involved in cellular processes, are also key to vector transmission [19,20,21,22]. A crucial role of the CP in non-persistent transmission by aphids has been proven for members of the genus *Potyvirus* and specific (DAG) motifs were identified as determinants for virus transmission [23,24,25]. In contrast, only little is known about semi-persistent virus transmissions, in which the virus is retained in the anterior foregut/cibarium of the vector [26]. Similar to the potyviruses, the CP appears to play a key role for virus acquisition and retention in the insect [27]. For the whitefly transmitted crinivirus lettuce infectious yellows virus (LYIV), the minor coat protein (CPm) was found critical for semi-persistent virus transmission. A frameshift mutation in the CPm leading to the expression of a truncated protein (211 vs. 453 aa) abolished WF transmission, while systemic movement of the virus was not affected [28].

Nothing is known about virus genes involved in *B. tabaci* vector transmission of members of the genus *Ipomovirus*, for which transmission efficiencies vary considerably among the species. CVYV and squash vein yellowing virus (SqVYV) are transmitted efficiently [11,18,29,30] while low transmission rates have been reported for tomato mild mottle virus (ToMMV) [31,32] and the viruses causing cassava brown streak disease (cassava brown streak virus and Ugandan cassava brown streak virus, U/CBSV) [33,34,35,36].

When comparing the otherwise diverse polyproteins of the *Ipomoviruses*, we noticed striking similarities between the CP of CVYV and that of the U/CBSV. Particularly, the core and carboxy-terminal regions of the CP were very similar (CVYV, aa 128–359) and presumably accounting for the observed serological cross reactions between U/CBSV and CVYV [37]. Considering the highly inefficient vector transmission of U/CBSV and a putative role of the *Ipomovirus* CP in the transmission process, we questioned whether the highly divergent N-termini (~38% aa identity) of the CP may explain the differences in transmission efficiencies between the viruses.

To study the role of the CP in WF transmission, we generated a collection of CP substitution and deletion mutants in an infectious cDNA clone of CVYV and analyzed their effect on vector transmission and pathogenesis. Our results demonstrate that the N-terminal region of the CVYV CP harbors amino acids involved in WF transmission, virus movement and symptom development in cucumber. Specifically, a deletion in amino acid position 93–105 (clone CVYV_CP_Del3) abolished vector transmission and resulted in delayed but more severe symptoms. Collectively, our data advance the knowledge about CVYV transmission and identify a CP region that plays a key role in the infection process.

## 2. Results

### 2.1. Generation of CVYV Mutant cDNA Clones

**Figure 2:**
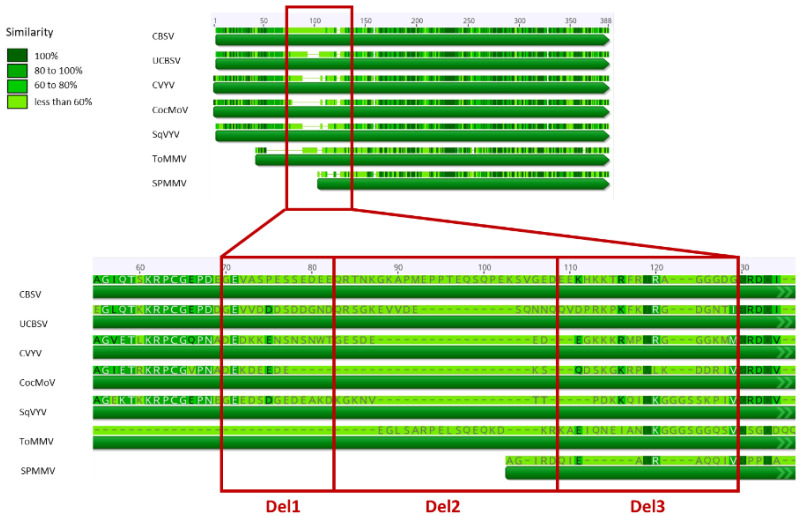
Graphical representation of the alignment of the coat protein of different species members of the genus *Ipomovirus*. The enlargement magnifies the hypervariable region that was selected for construction of deletion mutants CVYV_CP_Del1, CVYV_CP_Del2, CVYV_CP_Del3, coding for a CP carrying, respectively, a deletion of 10, 10 and 13 aa compared to the wild-type cucumber vein yellowing virus (CVYV) sequence. CBSV, cassava brown streak virus; UCBSV, Ugandan cassava brown streak virus; CocMoV, coccinia mottle virus; SqVYV, squash vein yellowing virus; ToMMV, tomato mild mottle virus; SPMMV, sweet potato mild mottle virus.

The alignment of CP amino acid sequences of *Ipomoviruses* revealed striking diversity in the N-terminal regions of the proteins (Figure 2). When comparing CVYV with the cassava *Ipomoviruses*, 38% aa identity was observed in position 1–128 (in the CVYV CP); a region of 33 aa in position 73–105 showed the highest variability (Figure 1B, gray region) and was therefore selected for mutagenesis. Three mutant clones (CVYV_CP_Del1, CVYV_CP_Del2, CVYV_CP_Del3) carrying, respectively, a deletion of 10, 10, and 13 aa in this region were constructed (Figure 1B and Figure 2, Table 1).

A 3D-modelling prediction of the CVYV CP revealed four motifs possibly exposed on the surface of the protein (Figure S1). The four motifs were located in the central region of the protein and their secondary structure was predicted to be a loop (motif 1 and 4), an α-helix (motif 2) and a loop/α-helix structure (motif 3). Amino acid residues located in these motifs were selected to generate 8 mutant clones, encoding a CP with double or triple amino acid substitutions (Table 1).

### 2.2. Effect of CP Mutations on Symptom Development and Severity

All constructs generated by alanine-scanning mutagenesis were infectious. However, only three alanine-substitution mutants (CVYV_CP_SQ, CVYV_CP_GV, CVYV_CP_QQ) were stable, while others reverted to the original sequence (Table 1). The mutants caused symptoms similar to the wild-type (wt), with the typical vein clearing appearing at approximately 12–14 days after inoculation (d.a.i.) (not shown).

**Figure 3:**
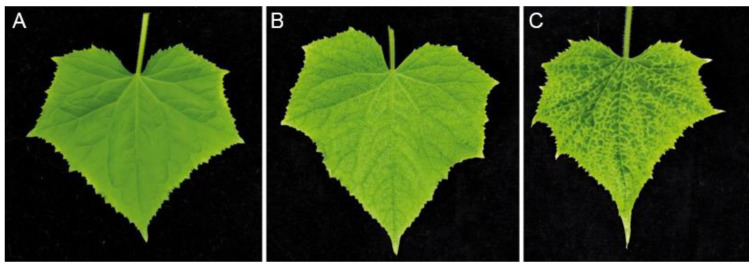
Comparison of cucumber leaf symptoms. (**A**) Healthy plant, (**B**) plant infected with wt CVYV and (**C**) plant infected with CVYV_CP_Del3, at 28 d.a.i.

Agroinfiltration of the deletion mutant clones CVYV_CP_Del1 and CVYV_CP_Del2, encoding a CP with a deletion of 10 aa at position 73–82 and 83–92, respectively, were not infectious, while the deletion mutant clone CVYV_CP_Del3 was infectious, and the introduced deletion was stable in successive mechanical transmission series. However, there was a delay of 7–10 days compared with the wt cDNA clone for symptoms to develop, and those became apparent ~21 d.a.i. In early infection stages, the symptoms resembled those of the wt, however during infection, vein yellowing symptoms became more severe (Figure 3) and extended to cover the entire leaf during late infections.

### 2.3. A Deletion in Position 93–105 in the N-Terminus of the CP Abolishes B. Tabaci Transmission

In whitefly transmission experiments, the three stable alanine-substitution mutants were transmissible, causing typical symptoms similar to the wt approximately 9–12 d.a.i. In contrast, CVYV_CP_Del3 was not transmissible and even high numbers of whiteflies and a prolonged acquisition- and inoculation access period (AAP/IAP) did not result in infections (Table 2). Absence of symptoms and negative RT-PCR tests confirmed loss of whitefly transmissibility in this otherwise very pathogenic virus mutant.

### 2.4. Two Partial Reversions in CVYV_CP_Del3 Restore Transmission

In order to fine map the CP region involved in whitefly transmission, the deletion region was shortened by inserting either eight or five amino acids into CVYV_CP_Del3 (Figure 1) to obtain CVYV_CP_Del3+8, CVYV_CP_Del3+5. Following agroinfiltration, the first symptoms appeared in *C. sativus* 14 d.a.i. for CVYV_CP_Del3+8, whereas CVYV_CP_ Del3+5 had a further delay of about five days. This was also evident when CVYV from agro-infected plants was mechanically transmitted to cucumber resulting in symptom phenotypes comparable to the wt (not shown). Furthermore, whitefly transmissibility was restored, and verification of the CP by direct sequencing of RT-PCR products confirmed the expected sequence.

## 3. Discussion

The genomes of viruses in the genus *Ipomovirus* differ from other genera within the family *Potyviridae.* While SPMMV and ToMMV still represent typical potyviruses, all other viruses lack HC-Pro, a critical gene with suppression of silencing function and involved in vector (aphid) transmission [38,39,40]. Instead, CVYV encodes a copy of P1, the P1b [14], to carry functions of an HC-Pro.

Transmission of *Ipomoviruses* by *B. tabaci* is in a semi-persistent manner, with transmission efficiencies varying largely with each virus species. The SPMMV was originally described as a whitefly-borne virus from Kenya, Uganda, and Tanzania [41], but later studies were unable to confirm its whitefly transmissibility [42]. For ToMMV, transmission assays also revealed ambiguous results [31,32,43,44]. However, using high numbers of whiteflies [31,32], *B. tabaci* was confirmed as vector of ToMMV. Whitefly transmission studies with CVYV isolates from Israel and Jordan showed plant infections already after an AAP as short as 30 min [11,18]. A transmission efficiency of 80% was reached with 30–35 WF per cucumber plant with AAP of 4 h and IAP of 24 h, respectively [18]. Transmission experiments of a Sudanese isolate from melon showed that virus acquisition was not efficient and the estimated probability of plant infections from transmission of single vector insects reached 0.088 after 48 h AAP [10]. Our experimental data showed an approximate 25% transmission efficiency with 4 h AAP and 2 h IAP using 10 WF per test plant. The efficiency dropped to ~15% when only 5 WF per plant were used for virus transmission (unpublished data). Whitefly transmission of CBSV was achieved with 20–25 WF per plant when AAP and IAP of 48h were given, resulting in an infection of 40% of the plants [35]. Our whitefly-transmission experiments with CBSV and UCBSV using herbaceous hosts revealed similar results [36]. However, they also showed that transmission was erratic and experimental reproducibility low, which led to our notion that a specific interaction between virus and its vector may have been lost for those viruses.

With *B. tabaci* being the insect vector for *Ipomoviruses*, a mechanistic explanation for its virus transmission however is still pending, and neither receptors in the vector nor motifs in the virus it transmits are known.

The surface-exposure of N- and C-termini of potyvirus CPs has been known for a long time [45], emphasizing the critical role of these regions in the interaction between virus and vector components. The N-terminus indeed harbors a DAG (Asp-Ala-Gly) motif, which is implicated in aphid transmission and was proven for many potyviruses [24,25,46,47,48,49,50]. Former studies have defined the N-terminus of potyviral CP as an “intrinsically disordered region”, presenting high sequence polymorphism and variable size, and being extremely flexible [22]. This characteristic has been associated to a functional versatility of this region, given the enormous plasticity which would allow multiple interactions and a role in different processes. The high diversity observed in the N-termini of otherwise highly similar CP sequences of CVYV and cassava brown streak viruses stimulated the hypothesis of a role of this region as determinant for whitefly transmission. We provided evidence that a deletion of 13aa in the N-terminus of the CP, at position 93–105, results in the loss of whitefly transmissibility. The alanine-substitution mutants introduced in the core region of the CP, predicted to be located in a small loop structure, were not stable and thus a critical role for amino acid residues located in loop structures of the CP, as described for other viruses [51,52], could not be studied. Indeed, similarly the CP of cucumber mosaic virus (CMV) was instable following mutagenesis of residues in loop secondary structures [53].

The restoration of transmissibility observed for virus constructs CVYV_CP_Del3+8 and CVYV_CP_Del3+5, supports that transmission competence was not assigned to a unique minimal motif in the Del3 CP region, as both reversions of eight and five amino acids in length resulted in transmissible viruses. Our results rather suggest that the CP region in position 93–105 is part of a structural domain that is necessary for interaction with the vector. Alternatively, disruption of the Del3 region could affect the folding in the N-terminus and prevent the correct assembly of CP structures required for transmission, as demonstrated in other virus/vector interactions [54,55]. Comparison of virus particle morphology by transmission electron microscopy revealed that wt CVYV and CVYV_CP_Del3 were indistinguishable, and virions with the expected morphology were assembled in both cases (Figure S2). Additional analysis of the morphology of viral-like particles (VLPs) composed of different CP versions expressed in heterologous systems could provide insights on the possible effects of CP aa substitutions on virion assembly and/or on structures required for vector interaction. A PVX-based viral vector for plant-based expression of VLPs of viruses with helical morphology, including CVYV, has been recently developed [56,57] and it could support further studies to understand the relevant role of specific amino acids.

A negative effect of CVYV_CP_Del3 infection on the behavior of the WF when feeding on infected plants was not evident by visual examination during the transmission experiments. In this context, a possible effect of virus-induced symptoms or alterations of the plant physiology on host attractiveness and vector feeding, as reported in other systems [58,59,60], does not fit with our observations.

The disruption of a putative vector-binding domain on the surface of the CVYV virion did not alter infectivity and movement functions of the capsid protein, since the construct CVYV_CP_Del3 was able to infect cucumber systemically. Mutants carrying a deletion in position 73–82 and 83–92 were not able to infect the plant and this correlates with the role of the N-terminal region of the CP of potyvirids in short- and long-distance movement [61,62,63,64]. CVYV_CP_Del3 was competent to move in its host plant. However, symptom development was delayed, showing that changes in this region affect the progression of symptoms.

The effect of amino acid substitutions in the viral CP on symptom expression has been documented for several viruses [64,65,66,67,68] and alterations in the secondary structure, rather than specific amino acid changes, were made responsible for it [69,70,71]. A modification of the secondary structure of CP appears to be associated with an increased symptom severity and this in turn is often correlated with an increased virus titer [72]. Compared to wt, CVYV_CP_Del3 mutant-infected plants showed delayed but more severe symptoms. This observation was, however, not further studied and thus conclusions on this aspect cannot be made.

Taken together, here we present evidence of the role of the N terminus of the CVYV CP for vector transmission and plant infection, and demonstrate that a region of 13 aa is required for whitefly transmission but is dispensable for systemic movement in the host plant. Further studies precisely revealing structural alterations of the CP and the localization of the virion in the vector can contribute to clarify the CVYV transmission process and provide insights into the puzzling transmission of other members of the genus.

## 4. Materials and Methods

### 4.1. CVYV cDNA Clone and Plant Material

An infectious cDNA clone of the CVYV isolate DSMZ PV-0776 [73], driven by the cauliflower mosaic virus (CaMV) 35S promoter in a pDIVA binary vector (GenBank acc. KX665539), was kindly provided by Dr. Edgar Maiß (Leibniz Universität Hannover, Hannover, Germany). Plants of *Cucumis sativus* var. Vorgebirgstraube were grown at 18 °C and kept at 22/23 °C for agroinfiltration, to be transferred after one week for maintenance at 25/26 °C.

### 4.2. Computational Analysis and Selection of Target Amino Acid Residues

An alignment of the CVYV CP sequence with the CP of members of the genus *Ipomovirus* was performed in Geneious Prime^®^ v. 2019.0.4 (Biomatters, Auckland, New Zealand) using the alignment program Clustal Omega, on selected species: *Cassava brown streak virus* (CBSV, NC_012698), *Ugandan cassava brown streak virus* (UCBSV, NC_014791), *Coccinia mottle virus* (CocMoV, AOC84052), *Squash vein yellowing virus* (SqVYV, NC_010521), *Tomato mild mottle virus* (ToMMV, NC_038929), *Sweet potato mild mottle virus* (SPMMV, NC_003797). The prediction of the secondary structure and solvent accessibility of the CVYV CP was performed using the protein modeling function of the Swiss-Model software (Swiss Institute of Bioinformatics, Lausanne, Switzerland) and superimposing on the *Potato virus Y* CP (source: RSCB Protein Data Bank, model 6HXX [74]).

### 4.3. Engineering of CVYV CP Mutants

For alanine-scanning mutagenesis of the selected residues, a plasmid carrying the 3′ region of the CVYV genome, including the CP ORF, was constructed. For this purpose, the full-length cDNA clone pCB_CVYV_VK representing the wild-type was used as template in a PCR reaction (Phusion Flash High-Fidelity PCR Master Mix kit; Thermo Fisher Scientific, Waltham, MA, USA) using primers CVYV_CP_F/CVYV_CP_Teilklon_R (Table S1). The PCR fragment was phosphorylated with T4 PNK Kinase (Thermo Fisher Scientific) and self-ligated, to generate clone pCB_CVYV_TK. The plasmid DNA was transformed into *Escherichia coli* strain DH5α, and plasmid DNAs were prepared from 3 mL overnight cultures using a Nucleospin Plasmid kit (Macherey-Nagel). Plasmid pCB_CVYV_TK was used as template to generate the desired CP mutant clones, using the primers reported in Table S1 and Phusion Flash High-Fidelity PCR Master Mix kit (Thermo Fisher Scientific). Each mutagenized CP was confirmed by sequencing and subsequently assembled into the full-length clone to replace the wt CP. Following, the CP mutants were amplified using primers CVYV_Gibson_CP_F/R and Phusion Flash High-Fidelity PCR Master Mix and assembled by Gibson Assembly (Gibson Assembly^®^ Master Mix, New England Biolabs GmbH, Germany) with two additional fragments obtained by PCR amplification of pCB_CVYV_VK using primer combinations CVYV_Open_3UTR_F/pDIVA_TrfA_R and CVYV_Open_NIb_R/pDiVA_TrfA_F. Mutant clones carrying a deletion in the N-terminus of the CP (pCB_CVYV_CP_Del1, pCB_CVYV_CP_Del2, pCB_CVYV_CP_Del3) were generated by Gibson Assembly using the full-length wt clone as template and primers reported in Table S1. The clones with a partially reverted CP (pCB_CVYV_CPDel3+8, pCB_CVYV_CPDel3+5) were generated by Gibson Assembly using as template plasmid pCB_CVYV_CP_Del3 and primers reported in Table S1. Gibson Assembly reactions were transformed in NEB^®^ 10-beta chemical competent *E. coli* cells according to the manufacturer′s instructions and bacterial colonies were grown for two days at 30 °C. Plasmids were purified from liquid cultures using the monarch plasmid miniprep kit (NEB). All generated constructs were verified by Sanger sequencing to proof read the desired mutation in the CP sequence.

### 4.4. Rhizobium Radiobacter Infiltrations and Mechanical Inoculations

Plasmids were transformed in *R. radiobacter* C58C1 cells, and infiltrated at OD600 nm = 0.8 in agroinfiltration buffer (MgCl2-Hexadydrat 10 mM, MES 10 mM, Acetosyringone 100 mM, pH 5.2) into the first true leaf of three week-old cucumbers. To favor infections, the needle of a syringe was used to prick the stem and inject the agrobacterium solution, while cotyledons were punctured and subsequently infiltrated using a 1 mL syringe. Mechanical inoculations were performed using sap obtained from infected leaf tissues ground in 0.05 M phosphate buffer, supplemented with carborundum and silica powders. The plants were grown up to four weeks post-inoculation to monitor symptoms development and for molecular tests.

### 4.5. RNA Extractions and RT-PCR of the CP Region

Total RNA from cucumber leaves was extracted using the RNeasy Plant Mini Kit (Qiagen, Hilden, Germany), according to manufacturer′s instructions. First-strand cDNA was synthetized using the SuperScript™ IV Reverse Transcriptase (Thermo Fisher, Waltham, MA, USA), with the specific reverse primer CVYV_RT_R (5′-TTTTATAACTTTACGCATAAAGG-3′). Two microliters of first-strand cDNA reaction were used for PCRs with primers CVYV_CP_F/R (Table S1) and the Taq DNA Polymerase kit (Invitrogen), to amplify the CP region. The RT-PCR products were analyzed by electrophoresis through a 1.0% agarose gels in TAE (Tris acetate EDTA) buffer. To confirm genome mutations, primers CVYV_CP_F/R were used for amplification reactions with the PhusionFlash High-Fidelity PCR Master Mix and the gel-purified PCR fragments (NucleoSpin Gel and PCR Clean-up kit, Macherey-Nagel, Germany) were directly sent for Sanger sequencing (Microsynth Seqlab, Göttingen, Germany). Sequence comparisons of CP sequences were carried out using Geneious Prime^®^ software (Biomatters, Auckland, New Zealand).

### 4.6. B. tabaci Transmission Assays

Non-viruliferous *B. tabaci* (MEAM 63) colonies were maintained on tomato and cucumber plants in insect-proof cages at 26 °C, with a 16/8 h light/dark period. For the WF transmission assays, at least ~50/60 WF adults were transferred onto a symptomatic leaf of an infected cucumber for an AAP of at least 4 h. Two healthy cucumbers at one true-leaf stage were used as recipient plants for an IAP of at least 4 h. WF were removed and the plants were monitored for symptom development for 30 days. The stable alanine substitution- and deletion mutants (CVYV_CP_SQ, CVYV_CP_GV, CVYV_CP_QQ, CVYV_Del3, CVYV_CP_Del3+5 and CVYV_CP_Del3+8) were tested for transmission, and the wild-type (CVYV_CP_wt) was used as control. To exclude a low transmission efficiency of CVYV_CP_Del3, a further transmission assay was conducted using ~150 WF with AAP and IAP on two recipient plants of 24 h each. Symptom development provided proof for whitefly transmission and virus infection, which was further verified by RT-PCR using primers CVYV_CP_F/R (Table S1).

## Figures and Tables

**Table 1:** List of the cucumber vein yellowing virus CP mutant clones. Position of amino acid substitutions and deletions affecting infection of *Cucumis sativus*.

Mutant Clone	Amino Acid Substitutions Position	Amino Acid Deletion Position	Residues Substituted into Alanine	Systemic Infection in *Cucumis sativus*
CVYV_CP_RD	142, 143	n.a.	RD	+°
CVYV_CP_SQ	171,172	n.a.	SQ	+
CVYV_CP_NW	175, 176	n.a.	NW	+°
CVYV_CP_TAS	180, 181, 182	n.a.	TAS	+°
CVYV_CP_GV	185, 186	n.a.	GV	+
CVYV_CP_QQ	189, 190	n.a.	QQ	+
CVYV_CP_LN	223, 224	n.a.	LN	+°
CVYV_CP_GE	227, 228	n.a.	GE	+°
CVYV_CP_Del1	n.a.	73–82	n.a.	−
CVYV_CP_Del2	n.a.	83–92	n.a.	−
CVYV_CP_Del3	n.a.	93–105	n.a.	+
CVYV_CP_Del3+8	n.a.	101–105	n.a.	+
CVYV_CP_Del3+5	n.a.	93–100	n.a.	+

° substitution not stable. n.a.: not applicable.

**Table 2:** Transmission experiments of wild-type and mutant CVYV with *Bemisia tabaci*.

Experiment	Clone	Number of WF/Test Plant	Infected Plants
1	CVYV_CP_wt	~60	2/2
2	CVYV_CP_wt	~20	2/2
3	CVYV_CP_wt	~30	2/2
4	CVYV_CP_wt	~30	2/2
5	CVYV_CP_wt	~30	2/2
6	CVYV_CP_wt	~30	2/2
7	CVYV_CP_wt	~40	2/2
8	CVYV_CP_wt	~40	2/2
9	CVYV_CP_SQ	~30	2/2
10	CVYV_CP_GW	~30	2/2
11	CVYV_CP_QQ	~30	2/2
12	CVYV_CP_Del3	~50	0/2
13	CVYV_CP_Del3	~50	0/2
14	CVYV_CP_Del3	~50	0/2
15	CVYV_CP_Del3	~50	0/2
16	CVYV_CP_Del3	~70	0/2 *
17	CVYV_CP_Del3+8	~40	2/2
18	CVYV_CP_Del3+8	~40	2/2
19	CVYV_CP_Del3+5	~40	2/2

Note: all experiments were conducted with AAP/IAP of at least 4 h; one experiment with clone. CVYV_CP_Del3 was conducted with 24h AAP/IAP (marked with *).

## Data Availability

The data presented in this study are available in article and supplementary material.

## Supplementary Materials

The following are available online at https://www.mdpi.com/article/10.3390/plants10122771/s1, Figure S1: (A) Modelling of the cucumber vein yellowing virus coat protein (CP), using the protein modeling function of the Swiss-Model software (Swiss Institute of Bioinformatics, Lausanne, Switzerland). The motifs targeted for site-directed mutagenesis are marked in color. (B) Position of the substituted amino acids in the CP amino acid sequence. The color of the motifs corresponds to the same one displayed in the model in panel A. Figure S2: Electron micrographs of cucumber vein yellowing virus (CVYV) particles from leaves of *Cucumber sativus* infected with the wild-type CVYV (A) and the CVYV_CP_Del3 mutant (B). Sap adsorption preparations were examined in a Tecnai G2 Spirit electron microscope (FEI Deutschland GmbH, Frankfurt, Germany), Table S1: Primers used for cucumber vein yellowing virus detection, alanine-scanning site-directed mutagenesis and Gibson assembly.
