# Supplementary material for: The Amino-Proximal Region of the Coat Protein of Cucumber Vein Yellowing Virus (Family Potyviridae) Affects the Infection Process and Whitefly Transmission
Source: Plants (Basel). 2021 Dec 15;10(12):2771. doi: 10.3390/plants10122771 (PMC8706179; doi:10.3390/plants10122771)
Supplement: Supplementary file 1 [file plants-10-02771-s001.zip › plants-1493215-supplement.pdf]

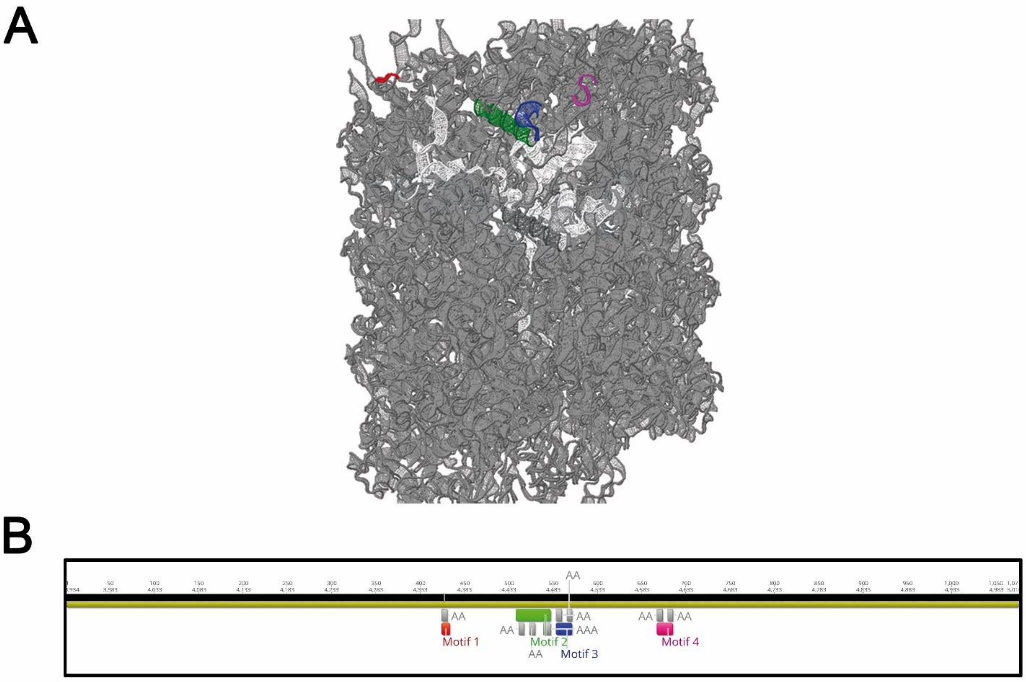

Figure S1. **A)** Modelling of the cucumber vein yellowing virus coat protein (CP), using the protein modeling function of the Swiss-Model software (Swiss Institute of Bioinformatics, Lausanne, Switzerland). The motifs targeted for site-directed mutagenesis are marked in color. **B)** Position of the substituted amino acids in the CP amino acid sequence. The color of the motifs corresponds to the same one displayed in the model in panel A.

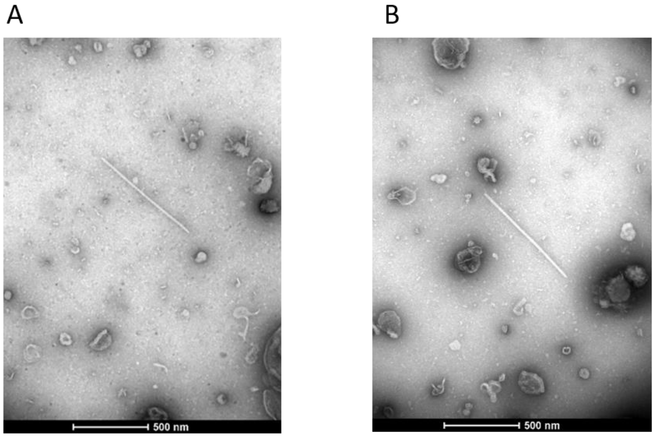

Figure S2. Electron micrograph of cucumber vein yellowing virus (CVYV) particles from leaves of infected *Cucumber sativus* infected with the wild-type CVYV (A) and the CVYV_CP_Del3 mutant (B). Sap adsorption preparations were examined in a Tecnai G2 Spirit electron microscope (FEI Deutschland GmbH, Frankfurt, Germany).
